# Preeclampsia and Blood Pressure Trajectory during Pregnancy in Relation to Vitamin D Status

**DOI:** 10.1371/journal.pone.0152198

**Published:** 2016-03-29

**Authors:** Linnea Bärebring, Maria Bullarbo, Anna Glantz, Monica Leu Agelii, Åse Jagner, Joy Ellis, Lena Hulthén, Inez Schoenmakers, Hanna Augustin

**Affiliations:** 1 The Department of Internal Medicine and Clinical Nutrition, Sahlgrenska Academy, University of Gothenburg, Gothenburg, Sweden; 2 Södra Älvsborgs Hospital, Department of Obstetrics and Gynecology, Borås, Sweden; 3 Department of Obstetrics and Gynaecology, Sahlgrenska Academy, University of Gothenburg, Gothenburg, Sweden; 4 Department of Antenatal Care, Primary Care, Närhälsan, Gothenburg, Sweden; 5 Department of Public Health and Community Medicine, Sahlgrenska Academy, University of Gothenburg, Gothenburg, Sweden; 6 Department of Antenatal Care, Primary Care, Närhälsan, Södra Bohuslän, Sweden; 7 MRC Human Nutrition Research, Nutrition and Bone Health Group, Cambridge, United Kingdom; Medical Faculty, Otto-von-Guericke University Magdeburg, GERMANY

## Abstract

Every tenth pregnancy is affected by hypertension, one of the most common complications and leading causes of maternal death worldwide. Hypertensive disorders in pregnancy include pregnancy-induced hypertension and preeclampsia. The pathophysiology of the development of hypertension in pregnancy is unknown, but studies suggest an association with vitamin D status, measured as 25-hydroxyvitamin D (25(OH)D). The aim of this study was to investigate the association between gestational 25(OH)D concentration and preeclampsia, pregnancy-induced hypertension and blood pressure trajectory. This cohort study included 2000 women. Blood was collected at the first (T1) and third (T3) trimester (mean gestational weeks 10.8 and 33.4). Blood pressure at gestational weeks 10, 25, 32 and 37 as well as symptoms of preeclampsia and pregnancy-induced hypertension were retrieved from medical records. Serum 25(OH)D concentrations (LC-MS/MS) in T1 was not significantly associated with preeclampsia. However, both 25(OH)D in T3 and change in 25(OH)D from T1 to T3 were significantly and negatively associated with preeclampsia. Women with a change in 25(OH)D concentration of ≥30 nmol/L had an odds ratio of 0.22 (p = 0.002) for preeclampsia. T1 25(OH)D was positively related to T1 systolic (β = 0.03, p = 0.022) and T1 diastolic blood pressure (β = 0.02, p = 0.016), and to systolic (β = 0.02, p = 0.02) blood pressure trajectory during pregnancy, in adjusted analyses. There was no association between 25(OH)D and pregnancy-induced hypertension in adjusted analysis. In conclusion, an increase in 25(OH)D concentration during pregnancy of at least 30 nmol/L, regardless of vitamin D status in T1, was associated with a lower odds ratio for preeclampsia. Vitamin D status was significantly and positively associated with T1 blood pressure and gestational systolic blood pressure trajectory but not with pregnancy-induced hypertension.

## Introduction

Every tenth pregnancy is affected by hypertension, one of the most common complications and leading causes of maternal death worldwide [1]. Hypertensive disorders in pregnancy include preexisting chronic hypertension, pregnancy-induced hypertension and preeclampsia (PE). PE is defined as hypertension (≥140/90 mmHg) and proteinuria, with onset after 20 weeks of gestation [2].

Approximately 2–7% of pregnancies are complicated by PE, depending on population and diagnostic criteria [3]. Risk factors for PE are nulliparity, multifetal gestation, previous PE, obesity and preexisting medical conditions such as chronic hypertension and diabetes [4]. PE is associated with increased maternal mortality and morbidity, e.g. pulmonary edema, eclampsia, renal or liver failure and stroke [3]. Moreover, studies suggest an increased risk of cardiovascular disease later in life for women having had PE [5]. Neonatal complications associated with PE include preterm delivery, intrauterine growth restriction, low birth weight and perinatal death [2]. In addition, low birth weight and growth restriction during fetal life are major risk factors for subsequent cardiovascular disease, according to the fetal origins of adult disease hypothesis [6]. Although the pathophysiology of the etiology of PE is unknown, abnormal placental development and associated placental hypoxia are believed to be primary causes [4]. Blood pressure (BP) during normal pregnancy initially decrease until mid-pregnancy when it begins to increase [7].

During the past decade, vitamin D status has been attributed health benefits beyond its recognized effects on bone health. For many of these, evidence of a causal relationship is lacking and RCT studies are sparse [8]. So far, controlled intervention trials sufficiently powered for PE are lacking [9], but one small study conducted reports no effects of vitamin D supplementation on PE [10]. Poorer vitamin D status (measured as 25-hydroxyvitamin D (25(OH)D) during pregnancy has been associated with increased risks of PE and gestational hypertension in some [11, 12] but not all observational studies [13, 14]. Cross sectional and cohort studies investigating the association between vitamin D status and PE show conflicting results [11–14]. Case-control studies often have insufficient or no matching, generating concerns about confounding as variations in vitamin D status associated with season, skin pigmentation and lifestyle factors are known [15]. Large case-control studies with covariate adjustment show inconsistent results [16–18]. In addition, case-control studies often include only one measurement of 25(OH)D, sometimes very late in pregnancy [19, 20]. This limits the evaluation of the potential role of vitamin D status during early pregnancy in the development and progression of PE. Also, reverse causality cannot be ruled out when 25(OH)D is measured after PE has developed.

The primary aim of this study was to test the hypothesis that there was an association between longitudinal 25(OH)D concentration during pregnancy and PE. Secondary aims were to test the hypothesis that there were associations between 25(OH)D concentration and gestational BP trajectory and pregnancy-induced hypertension.

## Materials and Methods

The GraviD study was conducted in connection with routine visits to the antenatal care in parts of the region Västra Götaland (Gothenburg, Södra Älvsborg and Södra Bohuslän) in southwestern Sweden, at latitude 57–58°N. The primary outcome of the study was to investigate the association between vitamin D status and hypertensive disorders in pregnancy and PE. The study design is a prospective population-based cohort study.

Recruitment took place during two time periods: fall 2013 (September 2^nd^- November 8^th^) and spring 2014 (February 24^th^- June 13^th^). During these periods, chosen to capture the seasonality of vitamin D status, all pregnant women registering at the antenatal care within the study areas were eligible for inclusion. The only exclusion criterion was pregnancy exceeding 16 gestational weeks at inclusion. Study information and consent forms were provided in eight languages to promote participation among many ethnic groups. Interpreters were employed if required, in line with standard antenatal care practice. This study was conducted according to the Declaration of Helsinki and all procedures were approved by the Regional Ethics Committee in Gothenburg. Written informed consent was obtained from all participants. Women who terminated their pregnancy, miscarried before gestational week 20 or were lost to follow-up (i.e. had moved) were excluded from analysis.

Blood samples were collected from each participant at two time points; before gestational week 16 (preferably at week 8–12, first trimester, T1) and after gestational week 31 (preferably at week 32–35, third trimester, T3), with gestational age determined by routine ultrasound. At both time-points, participants answered a questionnaire regarding lifestyle factors and background data. After delivery, medical records from antenatal care and obstetric departments were retrieved and data collected concerning BP, proteinuria, preexisting medical conditions, assisted reproduction, weight, height, employment status and tobacco use.

At the antenatal care, venous blood samples were drawn in gel serum separating tubes, centrifuged for 10 minutes within two hours of sampling and sent with regular laboratory transport to study personnel at Gothenburg University. Blood samples were kept from sunlight and refrigerated until and after transport. Study personnel extracted the separated serum; 56% of the samples were received within 12 hours, 59% within 24 hours and 95% within 36 hours. Only 5% of the samples were received after 36 hours and 1.7% after 48 hours. Serum was stored at -70°C until analysis of 25(OH)D. Previous studies have shown stability of 25(OH)D [21].

Laboratory analysis of total 25(OH)D was performed in batches and both samples from each woman were analyzed in the same batch. Analyses were performed by LC-MS/MS (Mass spectrometer API 4000) by the central laboratory at the University hospital in Malmö, Sweden certified by the Vitamin D External Quality Assessment Scheme. The LC-MS/MS method has a measuring range of 6–450 nmol/L for 25(OH)D3 and of 6–225 nmol/L for 25(OH)D2. The inter assay coefficient of variation is 6% at 40 nmol/L for both 25(OH)D3 and 25(OH)D2 [22]. All samples were analyzed for two fragments of 25(OH)D3 in order to increase measurement accuracy and decrease the risk of interference [22]. Serum 25(OH)D is the total of 25(OH)D2 and 25(OH)D3. Serum 25(OH)D2 was detectable in 14 women, and was not used separately in the subsequent analyses.

Systolic (SBP) and diastolic (DBP) blood pressure were measured as a part of standard practice of care, at baseline (T1) and within two weeks of gestational week 25, 32 and 37. PE was defined as at least two measures BP ≥140 or ≥90 mmHg after gestational week 20 and urinary protein ≥+1 on dipstick [23]. These measures were performed by midwives at the antenatal care, and recorded in the medical records. Medical records were reviewed by study personnel to confirm diagnoses as well as to find any undiagnosed cases of PE. Pregnancy-induced hypertension was defined as at least two measures BP ≥140 or ≥90 mmHg after gestational week 20, in previously normotensive women. Thus, women with preexisting hypertension could not be defined as cases and were excluded from analyses of PE and pregnancy-induced hypertension.

Weight was measured at the same visits as BP, according to standard procedure. Excessive gestational weight gain (GWG) was defined according to the BMI specific guidelines by the Institute of Medicine [24]. Preexisting medical conditions relevant to this study were heart disease, coagulations disorders including previous thrombosis, autoimmune disease (diabetes, multiple sclerosis, systemic lupus erythematosus and reumatoid arthritis) and kidney disease. Assisted reproduction was defined as either in vitro fertilization, intra cytoplasmic sperm injection or hormone therapy. Obesity at T1 was defined as BMI ≥30 kg/m^2^. Parity was defined as either nulliparous or parous and multifetal gestation as carrying more than one fetus. Origin was defined as being born in Northern Europe (yes/no) and employment status at baseline was categorized as unemployed/student, on parental leave, part-time employment or full-time employment.

Determinants of PE and pregnancy-induced hypertension were identified using logistic regression analysis and determinants of baseline BP were identified using linear regression analysis. In the analysis of determinants of PE, 25(OH)D variables included were continuous 25(OH)D at T1 and T3, and both continuous and dichotomous delta 25(OH)D. Delta 25(OH)D was calculated as the difference between 25(OH)D in T3 and T1, and was dichotomously coded as quartile 4 vs the lower three quartiles combined. Also, subgroup analysis of the women who conceived during Sept-Feb and March-Aug was performed for determinants of PE.

Differences in baseline BP between women with 25(OH)D above or below 50 nmol/L were evaluated using Student’s T-test. Changes in BP during pregnancy were evaluated using repeated measures ANOVA with post hoc Bonferroni. Determinants of BP trajectory during pregnancy were identified using linear mixed models analysis, separately for SBP and DBP. Two models were applied: 1) with BP at baseline, week 25, 32 and 37 in the repeated measures model and; 2) with BP at weeks 25, 32 and 37 while adjusting for baseline BP. These two models allows examination of the associations between BP trajectory and 25(OH)D besides baseline BP, and the longitudinal covariance between 25(OH)D and BP during pregnancy. In both these models, weight was used as a time-varying covariate (repeated measures for each time point). Vitamin D status was used as time-varying in model 1, where the mean of the 25(OH)D concentration in T1 and T3 was used as a proxy for vitamin D status at gestational week 25 and the concentration at week 32 (T3) was duplicated for week 37. In model 2, vitamin D status was fixed and only baseline 25(OH)D was included.

Data are presented as means and standard deviations (SD) unless otherwise stated. Analyses were adjusted for employment status at baseline, tobacco use at baseline, origin, calendar month at conception and gestational age at sampling. These variables were chosen as confounders on the basis of findings in other studies, and to enable comparisons.

Homogeneity of variance and normality of residuals for 25(OH)D was assessed using probability plots and box plots. Co-linearly was investigated using a correlation matrix and variables that had a correlation coefficient >0.7 was considered unfit for inclusion in the same model. Power calculations showed that a sample size of 2000 had 85% power to detect a doubled incidence of PE for women with serum 25(OH)D concentrations <25 nmol/l. Significance was accepted at p<0.05, and all p-values were two-tailed. Computer software IBM SPSS Statistics version 22.0 was used for all statistical analyses.

## Results

Of the 2126 women included in the GraviD-study, 120 miscarried or terminated their pregnancies and six were lost to follow-up. Thus, 2000 women were included in the analysis. In nine cases, serum from T1 was unsuitable for analysis, due to digression from the study protocol. Hence, 25(OH)D analysis was performed on 1994 samples from T1. In T3, 25(OH)D was measured in all 1834 samples that could be obtained. Samples at both T1 and T3 were available for 1827 women.

Blood samples at T1 and T3 were obtained at mean (SD) gestational week 10.8 (2.0) and 33.4 (1.8), respectively. At T1, participants had a mean age of 31 years and a mean BMI of 24.5 kg/m^2^. In total, 74% were born in Sweden and 75% in Northern Europe. At T1, 19% were unemployed and 60% of the women had studied at the university level (Table 1).

**Table 1:** Characteristics of participants.

	**Mean**	**Standard Deviation**	**N**
Age T1 (years)	31.3	4.9	2000
Height (cm)	166.8	6.3	1985
BMI T1 (kg/m^2^)	24.5	4.2	1972
25(OH)D T1 (nmol/L)	64.5	24.5	1994
25(OH)D T3 (nmol/L)	74.7	34.4	1834
Delta 25(OH)D^a^ (nmol/L)	10.2	30.4	1827
Gestational age T1 (weeks)	10.8	2.0	1994
Gestational age T3 (weeks)	33.4	1.9	1829
BP week 10 (mmHg) (SBP/DBP)	111.5/66.3	11.2/8.1	1861
BP week 25 (mmHg) (SBP/DBP)	111.9/65.0	11.3/7.7	1915
BP week 32 (mmHg) (SBP/DBP)	113.1/66.8	11.9/8.5	1923
BP week 37 (mmHg) (SBP/DBP)	116.1/70.6	11.8/8.9	1880
	**N (%)**		
Unemployed/student	367 (18.6)		
University-level education	1190 (59.8)		
Tobacco use at T1	89 (4.5)		
Born in North Europe	1504 (75.0)		
Nulliparity	836 (41.8)		
Excessive GWG^b^	653 (36)		
Preexisting medical condition^c^	58 (2.9)		
Preeclampsia	80 (4.0)		
Pregnancy-induced hypertension	160 (8.0)		

T1 = first trimester, BMI = body mass index, 25(OH)D = 25-hydroxyvitamin D, T3 = third trimester, BP = blood pressure, GWG = gestational weight gain

^a.^ Calculated as serum 25(OH)D at T3 minus 25(OH)D at T1.

^b.^ Defined as higher GWG than recommended in the BMI specific guidelines by the Institute of Medicine[24]. GWG calculated from T1 up until gestational week 37.

^c.^ Preexisting heart disease, coagulation disorder, kidney disease, diabetes, systemic lupus erythematosus, reumatoid arthritis.

Upon review of the medical records, 55 women had been diagnosed with PE by the antenatal care. Another 23 cases of PE were identified by the research team and two cases of gestational hypertension were reclassified as PE, in line with the study protocol. In total, 80 women developed PE, yielding an incidence of 4%.

Of the women who conceived during summer (June-Aug), 5.1% developed PE compared to 2.8% among women who conceived during winter (Dec-Feb). The incidence among women who conceived during spring (March-May) and autumn (Sept-Nov) was 4.0% and 2.1%, respectively. Fewer women conceived during spring and autumn, because of the windows of recruitment (201 and 48 conceived during spring and autumn, respectively).

Mean (SD) 25(OH)D concentrations were 64.5 (24.5) nmol/L in T1 and 74.7(34.4) nmol/L in T3. Mean delta 25(OH)D was 10.2 (30.4) nmol/L.

Results from logistic regression analysis of determinants of PE are shown in Table 2. In the adjusted multivariable analysis, multifetal gestation, nulliparity, baseline obesity, preexisting medical conditions and baseline DBP were positively associated with odds of PE, whereas 25(OH)D in T3 and delta 25(OH)D was negatively associated with odds of PE. Women with an increase in 25(OH)D ≥30 nmol/L (the highest quartile of delta 25(OH)D) had an odds ratio for PE of 0.22 (p = 0.002) in the adjusted multivariable model. Subgroup analysis of the 800 women who conceived during Sept-Feb showed a similar odds ratio for PE among women with a 25(OH)D increase ≥30 nmol/L (0.246, p = 0.026). There were no associations between PE and 25(OH)D among women who conceived during March-August (OR = 0.00, p = 0.997). T1 25(OH)D was not associated with PE in either bivariable or multivariable analysis.

**Table 2:** Bivariable and multivariable logistic regression analysis of the determinants of preeclampsia.

	**Bivariable analysis**					**Multivariable model**^a^				
	**B**	**OR**	**95% C.I.**		**P**	**B**	**OR**	**95% C.I.**		**P**
			**Lower**	**Upper**				**Lower**	**Upper**	
Delta 25(OH)D ≥30 (nmol/L)	-1.334	0.264	0.113	0.613	0.002	-1.510	0.221	0.084	0.581	0.002
Multifetal gestation	1.497	4.467	1.502	13.281	0.007	2.428	11.332	2.343	54.821	0.003
Obesity T1	1.514	4.544	2.758	7.486	<0.001	1.221	3.391	1.726	6.663	<0.001
Nulliparity	1.308	3.698	2.259	6.053	<0.001	1.432	4.188	2.279	7.697	<0.001
DBP T1	0.086	1.090	1.058	1.122	<0.001	0.089	1.094	1.055	1.134	<0.001
Preexisting medical condition T1	0.959	2.609	1.008	6.748	0.048	1.234	3.435	1.031	11.447	0.045
Excessive GWG	0.887	2.429	1.539	3.834	<0.001	0.517	1.677	0.951	2.958	0.074
Assisted reproduction	0.444	1.559	0.987	2.463	0.057	0.240	1.271	0.642	2.515	0.491
Age ≥40 years T1	-0.546	0.579	0.140	2.401	0.579	0.119	1.126	0.240	5.275	0.880
	**Bivariable analysis**					**Alternative multivariable models**				
	**B**	**OR**	**95% C.I.**		**P**	**B**	**OR**	**95% C.I.**		**P**
			**Upper**	**Lower**				**Upper**	**Lower**	
25(OH)D T1 (nmol/L)	0.004	1.004	0.995	1.014	0.354	0.004	1.004	0.991	1.016	0.571
25(OH)D T3 (nmol/L)	-0.005	0.995	0.988	1.002	0.173	-0.010	0.990	0.981	1.000	0.043
Delta 25(OH)D (nmol/L)	-0.009	0.991	0.982	0.999	0.028	-0.013	0.987	0.977	0.998	0.021

25(OH)D = 25-hydroxyvitamin D, T1 = first trimester, GWG = gestational weight gain, DBP = diastolic blood pressure, T3 = third trimester

Dichotomous: multifetal gestation, age ≥40 years, obesity, nulliparity, excessive GWG, preexisting medical condition, assisted reproduction and delta 25(OH)D ≥30 (4th quartile vs. quartiles 1–3).

^a.^Adjusted for 25(OH)D at T1, month of conception, gestational age at sampling, baseline tobacco use, Northern European birth country and employment status at baseline

SBP increased significantly from gestational week 25 (p<0.001), but there was no difference between weeks 10 and 25. DBP decreased significantly between gestational weeks 10 and 25 (p<0.001) and increased after week 25 (p<0.001). However, this decrease in DBP was not seen in women who were later diagnosed with PE (71 mmHg in week 10 and 73 mmHg in week 25, p = 1.00). In T1, women with 25(OH)D <50 nmol/L had lower SBP and DBP. This pattern was consistent through pregnancy (S1 Fig).

In the fully adjusted linear mixed model analysis, 25(OH)D concentration at T1 was positively related to SBP but not DBP trajectory (Table 3). In contrast, when baseline BP was not adjusted for, 25(OH)D trajectory was associated with DBP but not SBP trajectory (S1 Table). Both SBP and DBP trajectories were positively associated with non-obesity at baseline, nulliparity, higher weight trajectory and shorter height.

**Table 3:** Mixed models analysis of determinants of systolic (SBP) and diastolic blood pressure (DBP) trajectory during pregnancy, corrected for baseline BP^a^.

	SBP			DBP		
Adjusted^b^	Estimate	95% CI	P	Estimate	95% CI	P
25(OH)D T1 (nmol/L)	0.020	>0.00–0.04	0.02	0.009	<0.00–0.02	0.158
BMI ≥30 T1	-4.298	-5.80–-2.81	<0.001	-4.444	-5.60–-3.29	<0.001
Nulliparity	1.901	1.14–2.66	<0.001	1.903	1.32–2.49	<0.001
Preexisting medical condition T1	1.245	-0.07–2.56	0.063	0.812	-0.20–1.83	0.117
Age ≥40 years T1	0.682	-1.11–2.48	0.456	0.169	-1.22–1.55	0.811
Assisted reproduction	-0.792	-2.47–0.89	0.356	-0.613	-1.91–0.68	0.354
Height (cm) T1	-0.138	-0.20–-0.07	<0.001	-0.164	-0.21–-0.11	<0.001
Weight trajectory (kg)	0.278	0.242–0.31	<0.001	0.246	0.22–0.27	<0.001

SBP = systolic blood pressure, DBP = diastolic blood pressure, 25(OH)D = 25-hydroxyvitamin D, T1 = first trimester, BMI = body mass index. Reference categories for dichotomous variables are baseline BMI<30, parous, no preexisting medical condition, age <40 years, no tobacco use and no assisted reproduction. Weight, 25(OH)D and height are continuous.

^a.^ Blood pressure at 3 time points (gestational week 25, 32 and 37), adjusted for blood pressure at baseline (week 10)

^b.^ Adjusted for baseline SBP or DBP, multifetal pregnancy, Northern European birth country, baseline employment status, gestational age at baseline, month of conception and baseline tobacco use

In multivariable linear regression analysis, baseline SBP was positively related to obesity, nulliparity, age ≥40 years, height and 25(OH)D concentration at T1 (S2 Table). Baseline DBP was positively associated with obesity, nulliparity, preexisting medical conditions and 25(OH)D concentration at T1.

Pregnancy-induced hypertension was not associated with 25(OH)D at T1, T3 or with delta 25(OH)D in multivariable analysis (Table 4). Also, an increase in 25(OH)D ≥30 nmol/L was not associated with pregnancy-induced hypertension.

**Table 4:** Bivariable and multivariable logistic regression analysis of the determinants of pregnancy-induced hypertension.

	Bivariable					Multivariable^a^				
	B	OR	95% CI		P	B	OR	95% CI		P
			Lower	Upper				Lower	Upper	
25(OH)D T1	0.008	1.008	1.001	1.014	0.024	0.002	1.002	0.993	1.010	0.709
Obesity T1	0.366	1.443	0.897	2.321	0.131	-0.169	0.844	0.464	1.538	0.844
Nulliparity	0.361	1.435	1.039	1.983	0.029	0.205	1.228	0.854	1.765	0.269
Excessive GWG	0.473	1.604	1.146	2.246	0.006	0.363	1.438	0.995	2.078	0.054
Preexisting medical condition T1	0.632	1.881	0.876	4.040	0.105	0.089	1.093	0.454	2.629	0.842
Assisted reproduction	0.182	1.200	0.788	1.828	0.395	0.084	1.088	0.656	1.803	0.745
Tobacco use T1	-0.633	0.531	0.216	1.308	0.169	-0.447	0.640	0.262	1.563	0.327
Height	0.054	1.055	1.029	1.083	<0.001	0.035	1.035	1.005	1.066	0.021
Age ≥40 years T1	-0.141	0.868	0.373	2.024	0.744	-0.192	0.825	0.312	2.184	0.699
DBP T1	0.087	1.091	1.069	1.114	<0.001	0.088	1.092	1.067	1.117	<0.001

25(OH)D = 25-hydroxyvitamin D, T1 = first trimester, GWG = gestational weight gain, DBP = diastolic blood pressure,

Dichotomous: age ≥40 years, obesity, nulliparity, excessive GWG, preexisting medical condition and assisted reproduction

^a.^ Adjusted for multifetal pregnancy, baseline employment status, gestational age at T1, month of conception and northern European country of birth

## Discussion

These results are the primary outcome of the GraviD study–to our knowledge the first study reporting on the relationship between longitudinal vitamin D status and its relation to PE and gestational BP trajectory. Our results suggest that an increase of at least 30 nmol/L in 25(OH)D concentration during pregnancy is related to lower odds of PE, regardless of vitamin D status in early pregnancy. However, early pregnancy 25(OH)D concentration is positively related to baseline BP and to gestational SBP trajectory, although the associations are weak and their clinical significance may be questioned.

The concentration of 25(OH)D at T1 was not related to PE. There was a negative association between PE and 25(OH)D concentration at T3. Therefore, it is possible that vitamin D status in early pregnancy might not play a major role in placental development, but that an increment during gestation may prevent the development of PE. It has been suggested that 1,25(OH)_2_D influences the immunological tolerance during pregnancy and could play a role in the development of PE [25]. Our findings concur with a smaller cohort study that showed a tendency towards a decrease in 25(OH)D during pregnancy among women who developed PE while there was a slight increase in women who remained normotensive [12]. This difference was small and non-significant, possibly due to a smaller sampling interval since both samples were drawn in the second trimester. That study also found that lower 25(OH)D in late, but not early, second trimester was related to higher odds of PE [12]. Concurring with previous reports, we found that baseline obesity, nulliparity, multifetal gestation, preexisting medical conditions and BP at baseline was associated with PE [26].

The increment of at least 30 nmol/L in 25(OH)D concentration associated with lower odds of PE corresponds to the mean increment among women in this study who conceived during winter/spring, and is therefore a plausible estimate of the seasonal fluctuation in 25(OH)D concentration during pregnancy at northern latitudes. This seasonal effect also corresponds to that in a smaller pregnant Swedish cohort [15].

We found a positive association between vitamin D status and BP trajectory, but non-significant for DBP after adjustment for baseline BP. These results show that an increment in 25(OH)D of 1 nmol/L corresponds to an increase of 0.02 mmHg in SBP. We also found a positive association between 25(OH)D at T1 and SBP and DBP at T1. In it unclear if these associations are due to residual confounding or to a physiological mechanism. Further, the clinical relevance of this finding may be questioned as there were no associations between 25(OH)D and pregnancy-induced hypertension. Burris and colleagues [14] found a higher odds ratio for gestational hypertension with higher 25(OH)D at mean gestational week 28. Baseline DBP was the only significant determinant for both PE and pregnancy-induced hypertension, in multivariable analysis.

Limitations with our study are that BP data were obtained from medical records and not the results of standardized measurement. However, as this is expected to generate more variation, associations performed under standardized conditions may be stronger than shown here. Furthermore, all-year recruitment might have provided more detailed data on seasonal fluctuations of 25(OH)D in this population. The PE rate of 4% in the study sample is higher than the rate reported in Swedish national registry data (2.8% in 2013) [27]. This discrepancy might be due to undiagnosed PE, as we found that 29% of PE diagnosis was missing in the medical charts. If this is a reflection of all Swedish obstetric medical records, the actual national PE incidence might be closer to 3.6%. However, over-diagnosis of PE in the current study due to determination of proteinuria by dipstick cannot be ruled out. However, the preferred methods 24-hour urine collection or protein-creatinine ratio is not employed by antenatal care in screening for proteinuria [23].

Strengths of our study are that almost all pregnant women in Sweden attend the antenatal care [28], making it ideal for recruitment of a population-based cohort. Since about 1100 women register for antenatal care each month in the region, participation rate was roughly 32%, although it is unlikely that all eligible women were invited to participate due to substantial midwife workload. The study sample appears to resemble the general pregnant population in regard to education level, age, parity and origin [29]. Another strength is that blood samples were taken in both early and late pregnancy, enabling investigation of changes in vitamin D status in relation to PE and BP.

This is to our knowledge the first study to assess 25(OH)D concentration longitudinally during pregnancy, and its associations with PE, BP trajectory and pregnancy-induced hypertension. Our results show that an increment in 25(OH)D concentration during pregnancy is associated with lower odds of PE, regardless of early pregnancy vitamin D status. In multivariable analysis, 25(OH)D was not associated with pregnancy induced hypertension, despite a small positive association between early pregnancy 25(OH)D and SBP trajectory. In conclusion, an increase in 25(OH)D concentration during pregnancy of at least 30 nmol/L, regardless of vitamin D status at T1, was associated with a lower odds ratio for PE. Vitamin D status was positively associated with T1 BP and gestational SBP trajectory, but not with pregnancy-induced hypertension.

## Supporting Information

S1 Fig Unadjusted systolic (SBP) and diastolic blood pressure (DBP) trajectory in pregnancy, grouped by serum 25-hydroxyvitamin D (25(OH)D) at baseline (T1). Dashed line represents 25(OH)D <50 nmol/L and continuous line represents 25(OH)D ≥50 nmol/L. Error bars represent 95% CI.
(PDF)

S1 Table Mixed models analysis of determinants of systolic (SBP) and diastolic blood pressure (DBP) trajectory during pregnancy. (DOCX)

S2 Table Bivariable and multivariable linear regression analysis of the determinants of baseline blood pressure. (DOCX)
