# Supplementary material for: Preeclampsia and Blood Pressure Trajectory during Pregnancy in Relation to Vitamin D Status
Source: PLoS One. 2016 Mar 29;11(3):e0152198. doi: 10.1371/journal.pone.0152198 (PMC4811441; doi:10.1371/journal.pone.0152198)
Supplement: S1 Fig — Dashed line represents 25(OH)D <50 nmol/L and continuous line represents 25(OH)D ≥50 nmol/L. Error bars represent 95% CI. (PDF) [file pone.0152198.s001.pdf]

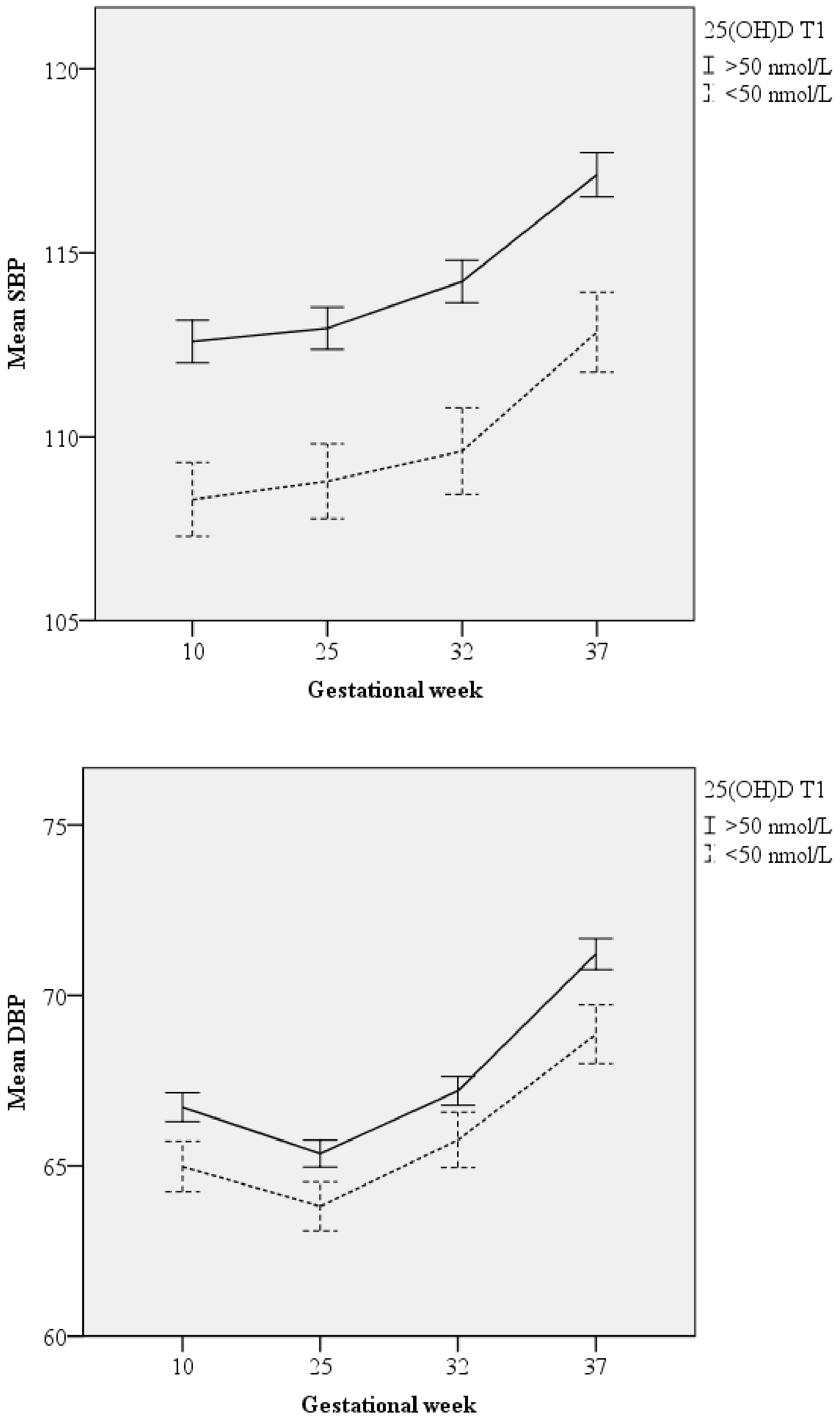

S1 Figure. Systolic (SBP) and diastolic blood pressure (DBP) trajectory in pregnancy, grouped by serum 25-hydroxyvitamin D (25(OH)D) at baseline (T1). Dashed line represents 25(OH)D <50 nmol/L (N=497) and continuous line represents 25(OH)D ≥50 nmol/L (N=1496). Error bars represent 95% CI.
