# Supplementary material for: Preeclampsia and Blood Pressure Trajectory during Pregnancy in Relation to Vitamin D Status
Source: PLoS One. 2016 Mar 29;11(3):e0152198. doi: 10.1371/journal.pone.0152198 (PMC4811441; doi:10.1371/journal.pone.0152198)
Supplement: S1 Table — (DOCX) [file pone.0152198.s002.docx]

S1 Table. Mixed models analysis of determinants of systolic (SBP) and diastolic blood pressure (DBP) trajectory during pregnancy^a^

|  | **SBP** | | | **DBP** | | |
| --- | --- | --- | --- | --- | --- | --- |
| **Adjusted**^b^ | **Estimate** | **95% CI** | **P** | **Estimate** | **95% CI** | **P** |
| 25(OH)D trajectory^c^ (nmol/L) | 0.007 | <0.00- 0.02 | 0.223 | 0.009 | >0.00- 0.02 | 0.047 |
| BMI ≥30 T1 | -2.794 | -4.27- -1.32 | <0.001 | -1.569 | -2.65- -0.49 | 0.004 |
| Nulliparity | 3.067 | 2.24- 3.89 | <0.001 | 2.202 | 1.61- 2.80 | <0.001 |
| Preexisting medical condition T1 | 1.619 | 0.18- 3.06 | 0.027 | 1.836 | 0.80- 2.88 | 0.001 |
| Age ≥40 years T1 | 1.992 | 0.06- 3.92 | 0.043 | 0.864 | -0.54- 2.26 | 0.226 |
| Assisted reproduction | 0.247 | -1.58- 2.07 | 0.791 | -0.441 | -1.76- 0.88 | 0.514 |
| Height (cm) T1 | -0.088 | -0.15- -0.02 | 0.009 | -0.134 | -0.18- -0.09 | <0.001 |
| Weight trajectory (kg) | 0.282 | 0.25- 0.31 | <0.001 | 0.205 | 0.18-0.23 | <0.001 |

1. Blood pressure at four time points at gestational week 10 (T1), 25, 32 and 37
2. Adjusted for multifetal pregnancy, Northern European birth country, baseline employment status, gestational age at baseline, month of conception and baseline tobacco use
3. 25(OH)D at gestational week 10 (measured), 25 (mean of samples in weeks 10 and 32), 32 (measured) and 37 (value from week 32 duplicated)
