# Supplementary material for: Preeclampsia and Blood Pressure Trajectory during Pregnancy in Relation to Vitamin D Status
Source: PLoS One. 2016 Mar 29;11(3):e0152198. doi: 10.1371/journal.pone.0152198 (PMC4811441; doi:10.1371/journal.pone.0152198)
Supplement: S2 Table — (DOCX) [file pone.0152198.s003.docx]

S2 Table. Bivariable and multivariable linear regression analysis of the determinants of baseline blood pressure

|  | **Bivariable analysis** | | | | **Multivariable analysis**^a^ | | | |
| --- | --- | --- | --- | --- | --- | --- | --- | --- |
|  | **SBP** | | **DBP** | | **SBP** | | **DBP** | |
|  | **β** | **P** | **β** | **P** | **β** | **P** | **β** | **P** |
| 25(OH)D (nmol/L) T1 | 0.07 | <0.001 | 0.03 | <0.001 | 0.03 | 0.022 | 0.02 | 0.016 |
| Obesity T1 | 4.20 | <0.001 | 3.90 | <0.001 | 4.91 | <0.001 | 4.26 | <0.001 |
| Nulliparity | 2.91 | <0.001 | 1.25 | 0.001 | 2.40 | <0.001 | 1.22 | 0.001 |
| Preexisting medical condition | 2.53 | 0.009 | 3.26 | <0.001 | 1.36 | 0.147 | 2.33 | 0.001 |
| Height (cm) T1 | 0.25 | <0.001 | 0.06 | 0.061 | 0.12 | 0.004 | 0.00 | 0.901 |
| Assisted reproduction | 1.53 | 0.206 | 0.33 | 0.708 | 1.00 | 0.401 | -0.22 | 0.802 |
| Age ≥40 T1 | 1.27 | 0.331 | 0.89 | 0.344 | 2.57 | 0.043 | 1.20 | 0.200 |

SBP= systolic blood pressure, DBP= diastolic blood pressure, T1= first trimester, 25(OH)D= 25-hydroxyvitamin D

Dichotomous: Obesity, nulliparity, preexisting medical condition, age <40 years, tobacco use and assisted reproduction. Continuous: Baseline 25(OH)D, weight and height.

1. Adjusted for baseline tobacco use, multifetal pregnancy, Northern European birth country, baseline employment status, gestational age at sampling, month of conception
